# Biological invasion and biological control select for different life histories

**DOI:** 10.1038/ncomms8268

**Published:** 2015-06-02

**Authors:** Ashraf Tayeh, Ruth A. Hufbauer, Arnaud Estoup, Virginie Ravigné, Léa Frachon, Benoit Facon

**Affiliations:** 1 UMR CBGP, INRA, 34000 Montpellier, France; 2 Department of Bioagricultural Science and Pest Management, Graduate Degree Program in Ecology, Colorado State University, Fort Collins, Colorado CO 80526-1177, USA; 3 UMR BGPI, CIRAD, 34000 Montpellier, France; 4 UMR Peuplements Végétaux et Bioagresseurs en Milieu Tropical, CIRAD-Université de la Réunion, Pôle de Protection des Plantes, 97410 Saint Pierre, France; 5 INRA, Laboratoire des Interactions Plantes-Microorganismes (LIPM), UMR441, F-31326 Castanet-Tolosan, France; 6 CNRS, Laboratoire des Interactions Plantes-Microorganismes (LIPM), UMR2594, F-31326 Castanet-Tolosan, France

## Abstract

Biological invaders have long been hypothesized to exhibit the fast end of the life-history spectrum, with early reproduction and a short lifespan. Here, we examine the rapid evolution of life history within the harlequin ladybird *Harmonia axyridis*. The species, once used as a biological control agent, is now a worldwide invader. We show that biocontrol populations have evolved a classic fast life history during their maintenance in laboratories. Invasive populations also reproduce earlier than native populations, but later than biocontrol ones. Invaders allocate more resources to reproduction than native and biocontrol individuals, and their reproduction is spread over a longer lifespan. This life history is best described as a bet-hedging strategy. We assert that invasiveness cannot be explained only by invoking faster life histories. Instead, the evolution of life history within invasive populations can progress rapidly and converge to a fine-tuned evolutionary match between the invaded environment and the invader.

## Abstract (web summary)

Understanding how biological invaders displace native species is challenging. Here, the authors compare the evolution of life-history strategies in the harlequin ladybird under laboratory conditions and show that invaders reproduce earlier and allocate more resources to reproduction than natives.

## Graphical abstract

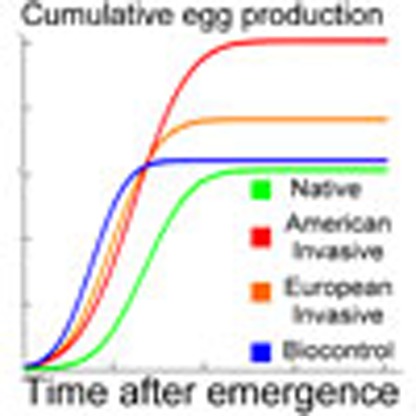

Ever since its beginning, invasion biology has struggled to understand how a species recently introduced into a new environment and selective regime can perform so well that it eventually displaces native species that are presumably locally adapted to that environment. Clearly, an advantageous combination of the timing of growth, reproduction and survival, or in the language of evolutionary ecology, an advantageous life-history strategy^1^, is necessary for successful invasion. A long-standing hypothesis is that invasive populations will display fast strategies^2^,^3^ along the fast–slow continuum^4^,^5^,^6^, which will be characterized by early reproduction, and a short lifespan. This idea is supported by several theoretical arguments. Fast life histories may be selected in risky environments where mortality, especially of juvenile stages, is high. Reproducing quickly also minimizes the amount of time spent at a demographically precarious small population size, and thus promotes successful invasion^2^. Lastly, in populations growing exponentially, as invasive populations may during the early stages of invasion, genotypes able to reproduce earlier than others are at an advantage^7^.

Cross-species comparisons provide an excellent overview of the kinds of life-history strategies likely to be linked to successful invasion. Although there is some empirical confirmation of the link between invasion and fast strategies from such studies^3^,^8^, it has become evident that a fast strategy is not consistently favoured during invasion^9^. For instance, in already occupied niches, higher competitive abilities could be selected for rather than higher fecundity^9^,^10^. Recently, a wide cross-species comparison in birds^11^ suggested that rather than a fast or slow strategy, a bet-hedging life history characterized by delayed reproduction and longer lifespan is linked to invasion.

Importantly, life histories can evolve rapidly^12^,^13^, and variation in life histories within species may occur between native and invasive populations. Furthermore, what is considered ‘fast' or ‘slow' is relative to other species and to the taxonomic group^14^. As such, a crucial next step to help put cross-species comparisons into deeper context is to understand, within a single species, how life history evolves during invasions. In particular, research on an individual species enables evaluating whether invasive populations evolve along the fast–slow continuum or shift towards other kinds of life histories when compared with native ones. Comparative work on life history between native and invasive populations within the same species provides evidence that in the absence of their usual enemies, plants can reallocate resources from defence to reproduction^15^, suggesting a rapid evolution of life history. However, there is remarkably little research tracking the entire reproductive life cycle of invasive species—especially of animals—hampering a thorough understanding of life-history evolution during invasions.

Here we describe the evolutionary changes in life-history strategies associated with the worldwide invasion of the harlequin ladybird *Harmonia axyridis*. This is a good study organism for examining rapid life-history evolution for several reasons. *H. axyridis* has a wide niche in its native area. The species is an opportunist predator, which is distributed from Siberia to South China and from Kazakhstan to Japan, covering an impressive range of climatic and ecological conditions^16^. Successfully invaded areas are also ecologically diverse. Invasion is recent (1988 for the outbreak in North East America and 2001 for the outbreak in Europe; with two to three generations per year, it respectively corresponds to 46–69 generations and 20–30 generations at the time of our sampling) and phenotypic differences between native and invasive individuals have evolved rapidly^17^,^18^. Invasive populations reach high densities and have become pests of small fruits as well as in houses, which they enter in autumn in an attempt to find suitable overwintering locations^16^. The species has long been used for classical biological control^19^, that is, purposefully introduced into a new range in an effort to impose top-down population regulation of pests, in particular aphids. European biocontrol individuals stem from populations reared in a predator-free environment and fed *ad libitum* for ∼100 generations^20^. Over the course of laboratory rearing, it is thus likely that they were unintentionally selected for high, early reproduction. Therefore, we hypothesize that they should exhibit a fast life-history strategy and consequently provide a positive control for comparison with native and invasive populations. There is no evidence of pure biological control strains being able to successfully found persistent populations in Europe.

The routes taken during the course of the invasion have been reconstructed meticulously^21^,^22^. These analyses have notably shown that a major North Eastern American invasive population was founded by individuals from the native area (Asia) and later acted as a bridgehead responsible for most other invasions worldwide. European invasive populations stem from admixture between the American invasive population and European biocontrol populations. This preliminary work enabled us to pinpoint two replicate populations within each of the four groups of interest, that is, native, American invasive, European invasive and biocontrol populations, while avoiding the well-known biases that can occur when inappropriate comparisons are performed^23^. By comparing the full adult life histories of different populations in a common environment, we precisely tested (1) whether there have been evolutionary shifts in life-history strategies between native and invasive populations, (2) whether biological control and invasion select for the same life history strategy and (3) how admixture between natural and biological control populations affects life-history strategies in the wild. While biocontrol populations clearly evolved towards the fast end of the life-history spectrum relative to native ones, invasive populations seem to have departed from this fast–slow axis with a reproduction spread over a longer lifespan. Life history can evolve rapidly over the course of an invasion but with a final outcome clearly depending on selection pressures imposed in the invaded environment.

## Results

### Biocontrol populations show fast strategies

The results reveal strong genetic differentiation in life-history strategies among *H. axyridis* populations. Specifically, all traits differ by origin (native, American invasive, European invasive and biocontrol; Supplementary Table 1). Within origin, no significant differences between populations were detected.

**Figure 1:**
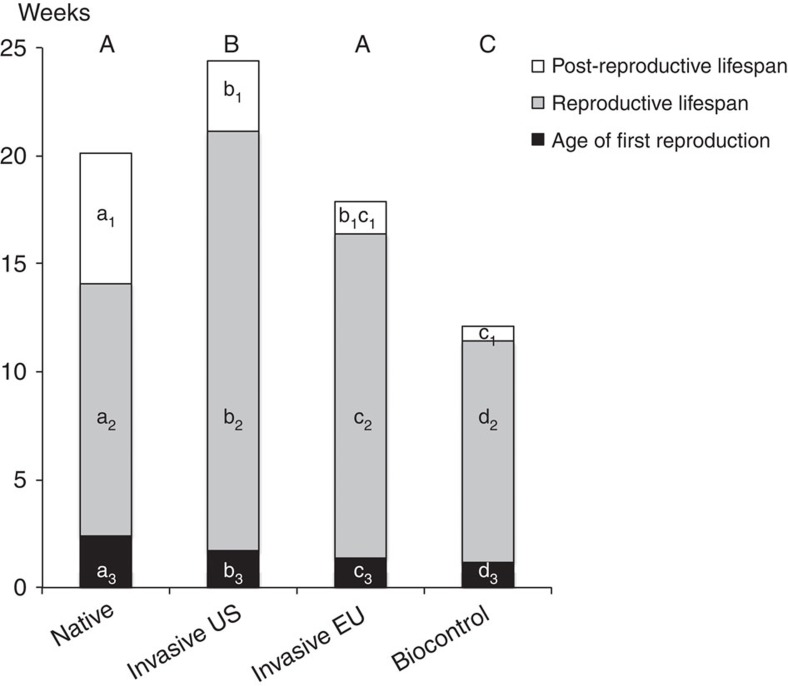
Reproductive schedules for the four different origins of populations. Mean time in weeks for age of first reproduction, reproductive lifespan, post-reproductive lifespan and adult lifespan for the four different origins of populations (native, American invasive, European invasive and biocontrol). Lower case letters represent significant differences within a life-history category, while capital letters indicate significant differences across the full adult lifespan.

**Figure 2:**
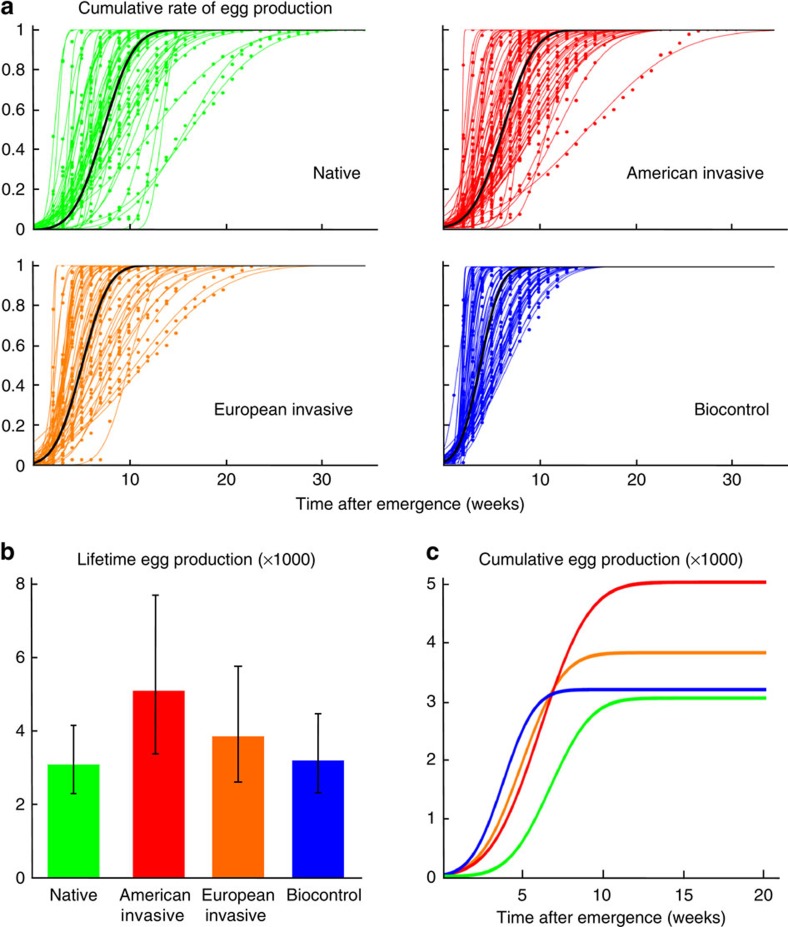
Lifetime dynamics of egg production in the four different origins of *H. axyridis*. (**a**) Temporal allocation of egg production. Coloured points and curves represent individuals' cumulative proportion of eggs produced over time. For each individual, data were fitted with the cumulative density function of a normal distribution with mean *μ* and standard deviation *σ*. Black curves were obtained using the average values of *μ* and *σ* in each type of population. (**b**) Mean lifetime egg production per type of population (with 95% confidence intervals). Data are from 242 individuals (*n*_Beijing_=27, *n*_Fuchang_=28; *n*_Chili_=27, *n*_USA_=40; *n*_Budapest_=25, *n*_Brussels_=26; *n*_Biotop_=42, *n*_Biobest_=27). (**c**) Schematic representation of lifetime dynamics of egg production within each type of population. The curves were obtained as the product of average allocation schedules (black curves in **a**) and average lifetime fecundities (**b**).

As we predicted, biocontrol individuals display fast strategies (Fig. 1, Supplementary Table 2), with a substantially shorter lifespan than those from other origins. As such, their reproductive lifespan is also shorter. Further, biocontrol females initiate reproduction earlier than those from other origins. Biocontrol females concentrate their egg production around a peak of fecundity that is both earlier and briefer than American invasive and native females (Fig. 2, Supplementary Tables 3, 5 and 7). Overall, biocontrol females have a higher average daily fecundity than those from the other origins, but due to their shorter lifespan their total fecundity is lower than American females and not different from the others (Supplementary Table 2).

### American invasive populations show bet-hedging strategies

American invasive females perform better than native ones for every measured trait. They have a longer total and reproductive lifespan than native ones (Fig. 1, Supplementary Table 2). They start reproducing earlier, and reach their fecundity peak earlier than native ones. American invasive and native females have an equivalent average daily fecundity, but because of their longer reproductive lifespan American invasive females have higher total fecundity (Fig. 2 and Supplementary Table 2). And yet, this strategy is very different from the typically fast one exhibited by biocontrol individuals. In contrast to the biological control populations, invasive *H. axyridis* produce their first eggs late and spread their egg production over a longer period (Fig. 2, Supplementary Tables 3, 5 and 7). This life history is best characterized as a bet-hedging strategy.

### Intermediate life histories in admixed invasive populations

Finally, the genetic admixture between American invasive and biocontrol populations have a long-lasting effect in the wild by shaping the life-history strategy of the European invasive individuals that result from this admixture. For most traits, age at the start of reproduction, total adult and reproductive lifespan, European invasive individuals display intermediate values between both parents (Figs 1 and 2, Supplementary Table 2). For the other traits, age at peak fecundity, daily and total fecundity, European invasive individuals share the same value as one of their parents (Figs 1 and 2, Supplementary Tables 2 and 3).

## Discussion

We show that life history can evolve rapidly over the course of an invasion, and that it is not necessarily a faster life history that evolves. In our focal species, the evolution of a fast life history is possible, as demonstrated by the dramatic shift in life-history strategy of populations raised in captivity in the context of biological control. Thus, it is not a constraint on life-history evolution that shaped the invasive populations, but rather responses to selection pressures in the introduced environment leading to a life-history strategy characterized by earlier reproduction and elongated reproductive lifespan.

Although biocontrol populations clearly evolved towards the fast end of the life-history spectrum as compared with native ones, invasive populations seem to have ‘escaped' from this fast–slow axis. Invasive populations did not only change the timing of offspring production, they exhibited higher fecundity than both native and biocontrol populations. Presumably, high fecundity, no matter what the timing of that fecundity, would be selected for in all environments, thus posing a puzzle of why it is evident only in the invasive populations.

One explanation lies in possible trade-offs with other traits not measured here: invasive individuals may have reallocated resources from other functions to reproduction. For instance, in research on plant invasions, there is evidence that higher performance of some invaders arises upon escape from their natural enemies^24^, and that as defenses against enemies are lost, an evolutionary shift of resources towards growth and reproduction occurs^25^,^26^. Although a similar shift might drive differences between native and invasive populations, it cannot explain the difference between biocontrol and invasive populations, as biocontrol populations live in a low-enemy environment. The same argument holds for many other attributes in which biocontrol populations have no need to invest, such as cold-tolerance or foraging abilities. Similarly, other work shows that populations from native and invasive origins do not differ in larval traits (Supplementary Fig. 4), so that a trade-off between larval and adult performances is unlikely to explain this increased lifetime fecundity.

A second possible explanation is that invasive individuals might have a higher rate of resources acquisition than the others^27^. A high rate of resource acquisition would be selected for when resources are reliable and abundant. As this is a feature of the environment experienced by biocontrol populations as well, it again does not explain why biocontrol individuals have lower fecundity than invasive individuals.

A last and more plausible explanation for the high fecundity of invasive individuals is reduced genetic load. There is good evidence that deleterious mutations were purged in the course of the *Harmonia* invasion^17^. This enables invasive individuals to exhibit higher fitness than native ones independent of the environment. This explanation could be further tested by quantifying the genetic load of biocontrol populations relative to native and invasive ones.

It is important to note that the European invasive populations show an intermediate life history, and intermediate fecundity between their parent populations (North American invasive and European biological control populations). This invasion is a more recent one than the American invasion, and thus it may be that a life-history strategy and high fecundity matching the American populations has yet to evolve. There are no quantitative data on whether European invasive populations are less invasive than American ones. Our results would predict, all else being equal, that European populations should pose less of a problem than North American populations do.

The high fitness, particularly of American populations, was found in conjunction with the evolution of the timing of offspring production, not towards a fast life-history strategy like seen in the biocontrol populations, but rather towards an elongated reproductive lifespan. These results match the pattern found in a global interspecific comparative analysis of avian introductions. To explain such pattern, Sol *et al*.^11^ argued that a long reproductive lifespan might more often be advantageous in the context of invasions than a fast life history. Prolonged reproduction may act as a bet-hedging strategy facilitating invasion success by reducing the risk of reproductive failure associated with maladaptation to a novel environment^11^. It may also facilitate establishment by means of other mechanisms such as the storage effect or by reducing population fluctuations. Determining whether the evolution of a long reproductive lifespan was instrumental in invasion success would require experimental releases, which is both unethical and in most locations illegal for predatory invasive species such as ladybirds. Additional research on the evolution of life-history strategies associated with invasions of other species would help determine if this pattern is general intraspecifically as it seems to be at the interspecific level^11^.

These results may also be useful in the context of biocontrol, and shed light on the nature of biological control. It is often assumed that classical biocontrol is a form of human-mediated invasion. But, when a period of laboratory propagation is involved, that may not be the case. In *H. axyridis*, selection in the laboratory has led not only to a shift in life history, but also to a higher susceptibility to pathogens, a lower rate of cannibalism rate^18^ and reduced survival at low temperatures^20^. Captive breeding practices may thus select for a suite of traits that could contribute to the failure of some biocontrol agents to establish well in the field, as indeed has been observed in this species^20^. Genetic drift may also partially explain changes in some of these traits, but it seems unlikely that drift alone is likely to account for the entire suite of changes. Response to laboratory selection is the more parsimonious explanation.

This study cements the fact that life history evolves rapidly, and is shaped by the particular environment experienced by populations, and thus could influence invasion success in critical ways. However, the ways that life histories shift during contemporary evolution seem more complex than previously thought, with a simple selection for a fast life-history strategy not found in all invasive populations.

## Methods

### Biological material and experimental design

Four different origins were considered: native, American invasive, European invasive and biocontrol. For each origin, two populations were included in the study. Fuchu in Japan and Beijing in China represented the native area. Two American invasive populations were sampled from Santiago in Chile and Brookings in USA. Two admixed invasive populations were sampled in Europe: Brussels in Belgium and Budapest in Hungary. Last, two commercial biological control populations from two different European biocontrol manufacturers (Biobest and Biotop) were also included. The genetic history among these populations has been described in detail^22^. All the eight populations were sampled between 2009 and 2011. Each population was maintained in the lab for two generations under strictly controlled conditions to minimize potential biases due to maternal and environmental effects before the experiment. Sixty G_3_ adults (1:1 sex ratio) per population were isolated just after emergence and monitored until death (480 individuals in total). Each female was presented with a male randomly chosen from the same population during 3 days every week leaving time for multiple copulations while minimizing density effects. Rearing conditions remained constant (24 °C, 60% RH; L:D 14:10) and individuals were fed *ad libitum* with irradiated eggs of *Ephestia kuehniella*. These conditions correspond to those used for the rearing of biocontrol individuals and are largely recognized as close to the species optimal conditions for growth, survival and fecundity. We followed individuals from their emergence as adults until death, recording egg production throughout, in a common laboratory environment. Phenotypic data on replicate individuals reared in a common environment reveal genetic differences in traits^28^. Thus, the experiment gave us data on genetically based differences among populations in key life history traits including total and reproductive lifespan, age at the start of reproduction, and total and daily fecundity.

### Statistical analyses

All traits that were directly measured (age at first egg production, average daily fecundity, total fecundity, reproductive and adult lifespan) were analyzed with mixed-model ANOVAs. Response variables were transformed when required to improve normality and reduce heteroscedasticity of the residuals. Models included origin (native, biocontrol, American invasive or European invasive) and sex (when required) as fixed effects, population nested in origin and block (the experiment has been split into two temporal blocks) as random effects (Supplementary Tables 1 and 2).

To better describe the temporal allocation of egg production, individual fecundity data (Supplementary Fig. 1) were transformed into a rate of egg production by dividing weekly egg production by each individual's total production. For each individual, the dynamics of the cumulative rate of egg production (p) over time was then fitted with the cumulative density function of a normal distribution with mean *μ* and standard deviation *σ* using nonlinear regression (Fig. 2a). Biologically, *μ* is the age at peak fecundity and *σ* quantifies the duration of high egg production (Supplementary Fig. 2, Supplementary Table 3). The distribution of both traits was analysed after proper transformation. The traits were positively correlated globally (Pearson's correlation coefficient *r*=0.813, *P*<0.0001, after log-transformation for *μ* and square-root transformation for *σ*, Supplementary Fig. 3), and within each origin (*r*=0.735 for native, *r*=0.814 for American invasive, *r*=0.892 for European invasive, *r*=0.939 for biocontrol females). For each trait, differences between origins were therefore investigated using ANCOVA with the other trait as independent variable and population origin as factor after Box–Cox transformation of the response variable and withdrawal of three outliers. For the duration of fecundity peak, *σ*, there was a significant interaction between the age at peak fecundity (*μ*) and population origin (Supplementary Fig. 3, Supplementary Table 4). We then focused on the comparison between pairs of populations using the same approach (Supplementary Table 5). For the age at fecundity peak, *μ*, the interaction between population origin and the width of fecundity peak (*σ*) was not significant (*P*=0.0804) and was therefore withdrawn from the model (Supplementary Table 6). As the interaction was not significant, we further looked for differences between pairs of populations using Tukey HSD tests (Supplementary Table 7).

## Additional information

**How to cite this article:** Tayeh, A. *et al*. Biological invasion and biological control select for different life histories. *Nat. Commun.* 6:7268 doi: 10.1038/ncomms8268 (2015).

## Supplementary Material

Supplementary Information Supplementary Figures 1-4 and Supplementary Tables 1-7
