# Supplementary material for: Biological invasion and biological control select for different life histories
Source: Nat Commun. 2015 Jun 2;6:7268. doi: 10.1038/ncomms8268 (PMC4458860; doi:10.1038/ncomms8268)
Supplement: Supplementary Information — Supplementary Figures 1-4 and Supplementary Tables 1-7 [file ncomms8268-s1.pdf]

Supplementary Figure 1. Lifetime dynamics of egg production: raw data. Each line joins the number of egg produced weekly by one individual, until its death.

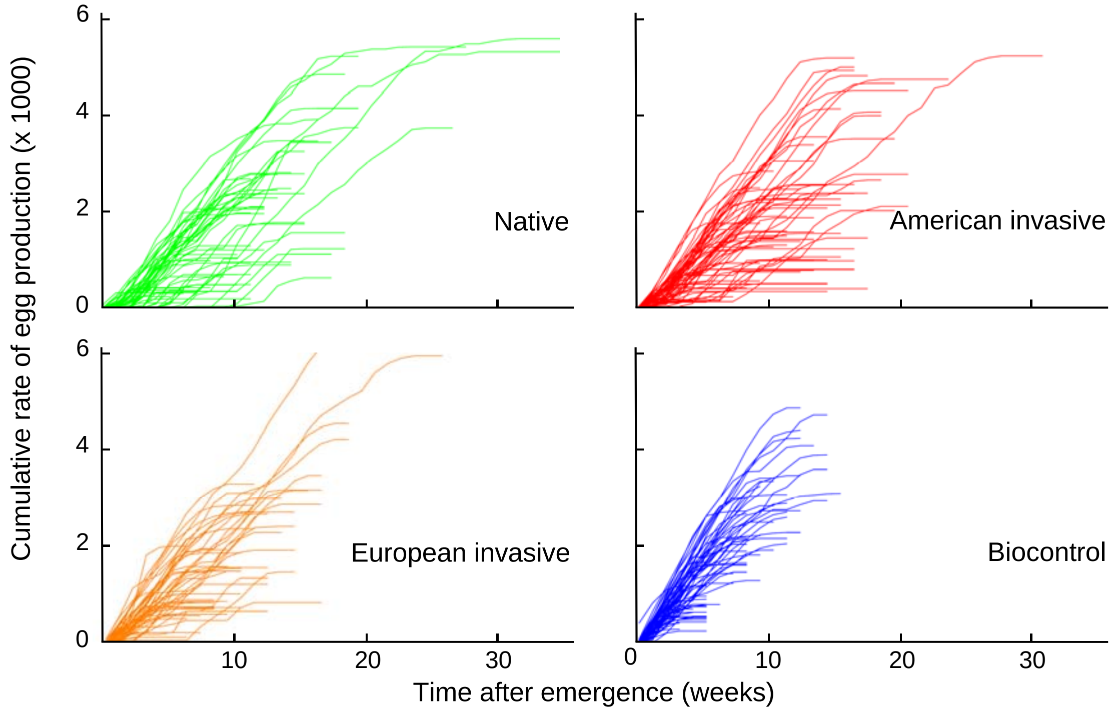

Supplementary Figure 2. Mean rate of egg production per week for the four types of populations.

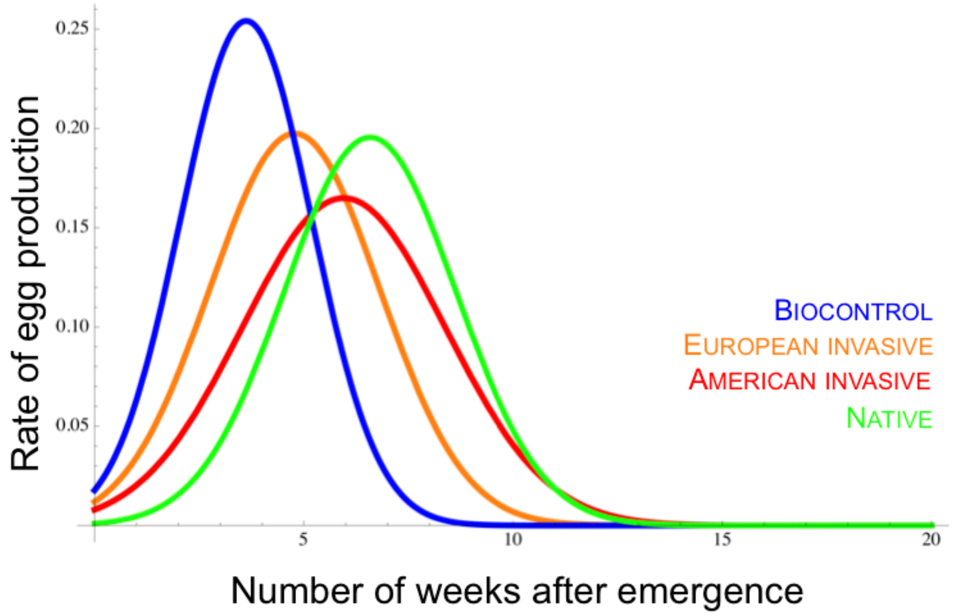

Supplementary Figure 3. Relationship between the age at and duration of fecundity peak in the four types of populations. B = Biocontrol, A = European invasive, P = American invasive, N = Native.

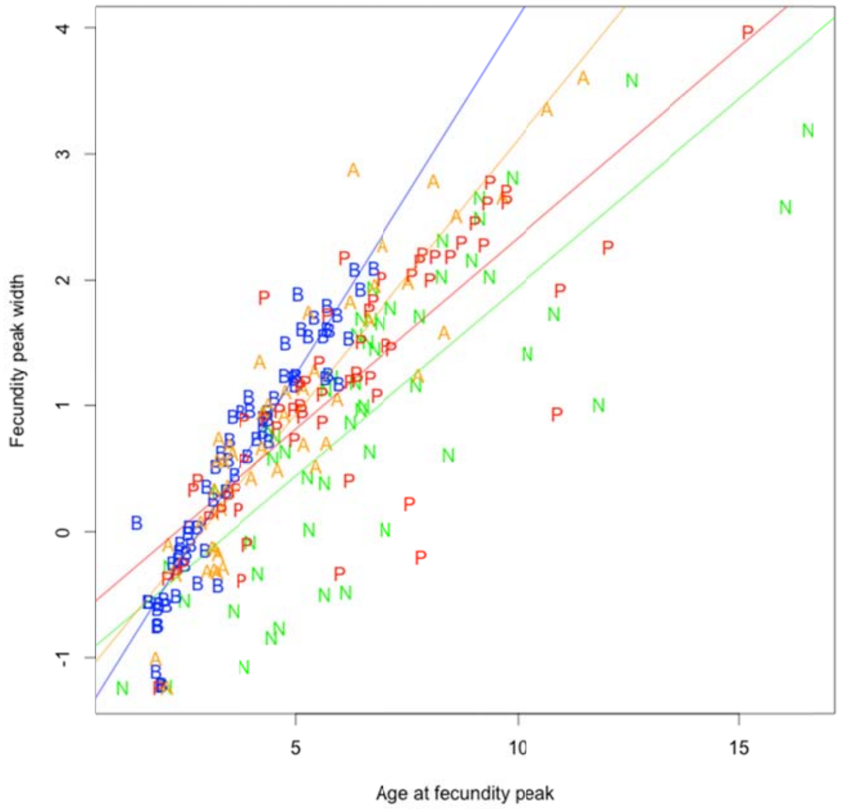

Supplementary Figure 4. Mean larval traits for native and invasive populations (with 95% confidence intervals). Triangle represent hatching rate and rectangle represent larval survival. Data are retrieved from Facon B. *et al.* Inbreeding depression is purged in the invasive insect *Harmonia axyridis*. *Curr. Biol.* **21**, 424–427 (2011).

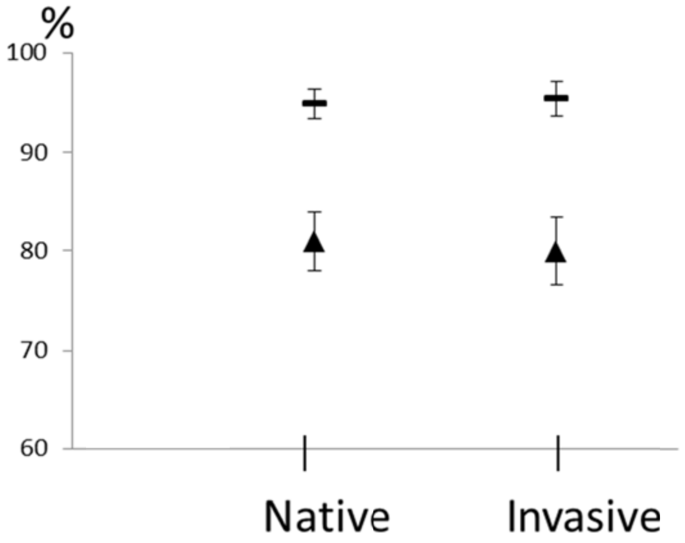

Supplementary Table 1. Details of statistical analyses for the different studied traits.

[1]Origin in all models is treated as a fixed effect, and tested with type III *F* statistics, with denominator degrees of freedom calculated using either the Satterthwaite or kr approximation.

[2]Population(origin) and block are treated as random effects in all models, and are tested with likelihood ratio tests in which the test statistic is distributed as X^2. These tests have one degree of freedom because they are one-sided.

Source	Test statistic	d.f.	P-value
**Average daily fecundity**			
Origin[1]	5.17	3, 75.9	0.003
Population (origin)[2]	0.1	1	0.38
Block[2]	0	1	0.5
**Total fecundity**			
Origin	4.67	3, 238	0.003
Population (origin)	17.2	1	0.00002
Block	6.8	1	0.005
**Age of first reproduction**			
Origin	35.83	3, 236	< 0.0001
Population (origin)	0	1	0.5
Block	0	1	0.5
**Reproductive lifespan**			
Fixed effect			
Origin	10.4	3, 236	< 0.0001
Population (origin)	14.2	1	0.00008
Block	9.8	1	0.0009
**Adult lifespan**			
Fixed effect			
Origin	36.4	3, 3.88	0.003
Population (origin)	0	1	0.5
Block	12.9	1	0.0002

Supplementary Table 2. Means and 95% CI for the measured traits.

	Average daily fecundity (# eggs)	Lifetime egg production (# eggs)	Age of first reproduction (weeks after emergence)	Reproductive lifespan (weeks)	Adult lifespan (weeks)
Native	35.3 (31.3-39.8)	3033.0 (2253.8-4081.6)	2.4 (2.1-2.6)	11.7 (9.1-15.1)	17.7 (15-20.8)
Invasive US	33.6 (29.0-39.0)	5001.5 (3319.2-7536.6)	1.7 (1.5-1.9)	19.4 (13.6-27.6)	22.7 (17.9-28.8)
Invasive EU	33.5 (28.9-38.8)	3802.1 (2570.1-5624.6)	1.4 (1.3-1.6)	15 (10.7-20.9)	16.5 (13.2-20.6)
Biocontrol	42.0 (37.2-47.4)	3178.6 (2310.7-4372.5)	1.2 (1-1.3)	10.2 (7.7-13.4)	10.9 (9.1-13.0)

Supplementary Table 3. Means and 95% CI for the traits estimated from individual nonlinear regression over individual egg production timing for the four different origins.

	Duration of fecundity peak (σ) in weeks	Age of peak fecundity (μ) in weeks
Native	2.04 (1.65-2.47)	6.58 (5.77-7.44)
Invasive US	2.42 (2.08-2.79)	5.95 (5.33-6.60)
Invasive EU	2.02 (1.64-2.43)	4.78 (4.19-5.41)
Biocontrol	1.57 (1.32-1.84)	3.62 (3.28-3.98)

Supplementary Table 4. ANCOVA summary for boxcox(σ) ~μ*pop.

Response variable: σ	Df	SumSq	MeanSq	F value	Pr(>F)	
μ	1	169.45	169.45	599.79	<2e-16	***
Origin	3	11.85	3.95	13.98	2.17e-08	***
μ:origin	3	11.01	3.67	12.99	7.38e-08	***
Residuals	225	63.57	0.28			

Supplementary Table 5. *P*-value of factor origin for duration of fecundity peak, σ, in an ANCOVA conducted on pairs of origins.

Pair of populations	P-value of factor origin	P-value of interaction
Native-American invasive	0.0038	NS
Biocontrol – Native	< 0.0001	< 0.0001
Biocontrol – European invasive	0.0047	0.0045
Biocontrol – American Invasive	0.0046	< 0.0001

Supplementary Table 6. ANOVA table for boxcox(μ)~σ+pop on the whole dataset.

Response variable: μ	Df	Sum Sq	Mean Sq	Fvalue	Pr(>F)	
σ	1	112.34	112.34	743.92	<2e-16	***
factor(pop)	3	13.76	4.59	30.37	<2e-16	***
Residuals	228	34.43	0.15			

Supplementary Table 7. Multiple mean comparisons between population origins using Tukey procedure.

	Difference	Lower bound	Upper bound	P-value
EurInv-Bio	0.1582013	-0.03025571	0.3466584	0.1340396
AmerInv-Bio	0.3903685	0.21450210	0.5662349	0.0000002
Nat-Bio	0.6298793	0.44560179	0.8141567	0.0000000
AmerInv-EurInv	0.2321672	0.04060372	0.4237306	0.0103645
Nat-EurInv	0.4716779	0.27236472	0.6709911	0.0000000
Nat-AmerInv	0.2395108	0.05205761	0.4269639	0.0059969
